# Fusion of classical and deep learning features with incremental learning for improved classification of lung and colon cancer

**DOI:** 10.1038/s41598-025-24734-w

**Published:** 2025-11-19

**Authors:** Mullakuri Anusha, D. Srinivasulu Reddy

**Affiliations:** 1 Department of ECE, Jawaharlal Nehru Technological University Anantapur, Anantapuramu, India; 2 Department of ECE, Sri Venkateswara College of Engineering, Tirupati, Affiliated to Jawaharlal Nehru Technological University Anantapur, Anantapuramu, India

**Keywords:** Lung cancer, Colon cancer, Histopathology, Transformer fusion, Incremental learning, Imaging, Software, Cancer, Cancer, Medical research, Oncology, Cancer

## Abstract

Correct histopathological image classification of lung and colon cancer is a stringent challenge for clinical pathology. This work introduces a hybrid deep learning network by combining traditional handcrafted features of LBP, GLCM, wavelet, color, and morphological descriptors with deep features derived from an extended EfficientNetB0. A transformer-based attention fusion strategy is adopted to fuse these heterogeneous representations, facilitating robust multi-scale feature learning. To even better accommodate adaptability and curtail catastrophic forgetting, the model is trained with an adaptive incremental learning approach with stage-wise data augmentation. The suggested method is trained on the LC25000 dataset and tested on two public, independent datasets, NCT-CRC-HE-100K and HMU-GC-HE-30K, showing consistent performance with accuracies of 99.87%, 99.07%, and 98.4%, respectively. These findings are affirmations of the framework’s generalizability, scalability, and clinical applicability in multi-class histopathological image classification. All source code and dataset access instructions are publicly made available to encourage reproducibility and extension.

## Introduction

Lung and colon cancers are two of the most significant causes of cancer morbidity and mortality globally, which require accurate and timely diagnosis to treat them effectively. Histopathological evaluation of tissue specimens remains the mainstay of cancer diagnosis, which allows pathologists to examine cellular features, such as nuclear atypia, mitotic activity, and architectural arrangement, that are crucial for classification and staging. Recent developments have witnessed the incorporation of computer-assisted diagnostic (CAD) systems that employ sophisticated image processing algorithms so that histopathological slides can be analyzed automatically. Methods like digital pathology, in which whole-slide imaging (WSI) is used, make it possible to quantify histological features. In addition, the use of deep learning algorithms for image analysis has shown fruitful outcomes for the discrimination of cancerous and non-cancerous tissues, thus improving diagnostic precision and workflow efficiency in a clinical environment.

Most studies have emphasized how to improve the classification accuracy of lung and colon cancers using various sophisticated methods. Hybrid model integrating GoogLeNet, VGG-19, and Principal Component Analysis (PCA) with Artificial Neural Networks (ANN)^1^. This multi-dimensional model exhibited an extremely high accuracy rate by successfully extracting and performing dimensionality reduction from the LC25000 dataset. Few researchers implemented an ensemble approach using MobileNet, InceptionV3, and VGG16, which allowed for better generalization through the exploitation of the strengths of the various architectures^2^. The application of ensemble methods reflects the benefit of aggregating different models to extract various features and representations in histopathology images.

One researcher took it a step further by investigating multi-modal image processing based on resilient neural networks. The focus is on combining different imaging modalities, which can complement each other to contribute to a robust classification by providing more information^3^. The Use of ResNet50 and EfficientNetB0 leads to model complexity and significant requirements for computational power, especially in settings with limited processing capacity^4^. An author used optimized deep neural networks but observed that the performance of their model was highly variable across various datasets, which showed poor generalization of the model^5^. DeepHistoNet was proposed, which was good at the LC25000 dataset but was plagued by overfitting, which made its usability in a clinical environment where variability in data is a routine suspect^6^. Usage of CNNs in conjunction with other models, including LightGBM and EfficientNetB0, but their dependence on single-model-based methods restricted their flexibility to the varied features of histopathological images^7,8^.

Most existing methods for lung and colon cancer classification tend to be based on single-model techniques or do not employ a wide variety of feature extraction methods, which reduces their generalizability and robustness across datasets. Moreover, overfitting due to model complexity and decision-making lack of interpretability also restricts their clinical usability. The proposed approach overcomes these difficulties by applying a complete classical feature extraction process in combination with a revised EfficientNetB0 model and a transformer fusion mechanism. This not only integrates dissimilar features from classical and deep learning models but also utilizes a 5-fold cross-validation approach and incremental learning to increase model robustness and prevent overfitting. Through the use of the strengths of multiple types of features and strict validation, the proposed approach substantially enhances classification accuracy while preserving interpretability, making it more clinical-friendly.

The LC25000 dataset is a valuable resource created for the testing and development of machine learning models for medical image classification, specifically cancer detection. Containing 25,000 high-resolution images, the dataset is split into five different classes as specified in Table 1: colon_aca (colon adenocarcinoma), colon_n (normal colon), lung_aca (lung adenocarcinoma), lung_n (normal lung), and lung_scc (lung squamous cell carcinoma). Each of the five classes has 1500 images, making the dataset well-balanced and supporting model training and validation across different types of cancers. The LC25000 dataset has been rigorously tested and validated, with its images handpicked to improve quality and relevance to medical image classification applications. The dataset has been employed in some research studies and benchmarks, which have proven its dependability and effectiveness in training machine learning models for cancer detection^9^. The images contain varying textures and color patterns typical of real clinical samples, thereby supporting the creation of strong diagnostic tools that can effectively generalize to real-world medical cases^10^.

Recent studies have explored hybrid feature fusion approaches that combine handcrafted and deep learning representations for medical image classification. Notably, the CancerNet framework that combines radiomics and deep features from a pre-trained AlexNet to differentiate gastrointestinal stromal tumors (GIST) from CT imaging was proposed^11^. However, this study is limited to binary classification on a relatively small dataset and does not accommodate incremental learning or attention-based fusion. Along the same lines, fusion framework of radiomics and deep learning to differentiate breast cancer lung metastasis (BCLM) and primary lung cancer (PLC) using low-dose CT was proposed^12^. While their use of intra and peri tumoral features is new in CT imaging, their model is domain-specific, lacks interpretability mechanisms such as attention, and is limited to binary classification.

On the contrary, our proposed framework is specifically designed for multi-class histopathological image classification of both lung and colon cancer using H&E-stained slides of the public large-scale dataset LC25000. It is tested on two external public datasets, NCT-CRC-HE-100K and HMU-GC-HE-30K. We employ a variant EfficientNetB0 backbone for deep feature extraction with fewer parameters and better representation. Conventional features like LBP, GLCM, wavelet, and morphological descriptors are appended to extract domain-specific structure and texture. Moreover, our use of morphological features like area, eccentricity, solidity, and extent provides structural and geometric information about cellular organization, which is critical in histopathological grading but was not utilized in the aforementioned works. These heterogeneous features are fused with a novel transformer-based attention mechanism that learns inter-feature importance dynamically, in contrast to static concatenation in earlier works. Furthermore, our method supports adaptive incremental learning, allowing batch-wise training that mimics real-world data accumulation and avoids catastrophic forgetting, an essential step toward clinical deployability.

**Table 1:** LC25000 dataset images distribution.

Class	Number of images	Description	Image properties
Colon Adenocarcinoma	5,000	Tumor tissues from colon patients	High-resolution, RGB
Normal Colon	5,000	Healthy colon tissues	High-resolution, RGB
Lung Adenocarcinoma	5,000	Tumor tissues from lung patients	High-resolution, RGB
Normal Lung	5,000	Healthy lung tissues	High-resolution, RGB
Lung Squamous Cell Carcinoma	5,000	Tumor tissues from lung patients	High-resolution, RGB

To effectively utilize the LC25000 dataset for the proposed work, each class consisting of 5,000 images was divided into five stages, with each stage containing 1,000 images. The initial training was conducted using the first 1,000 images from each class, establishing a foundational model. This was followed by incremental training phases, where the subsequent 1,000 images were used in each training stage as given in Table 2. This structured approach allows the model to gradually learn from the additional data while retaining knowledge from previous stages, thereby enhancing its performance and adaptability.

**Table 2:** Image splitting for incremental training stages.

Colon Adenocarcinoma	Normal Colon	Lung Adenocarcinoma	Normal Lung	Lung Squamous Cell Carcinoma	Total Images
1,000	1,000	1,000	1,000	1,000	5,000
1,000	1,000	1,000	1,000	1,000	5,000
1,000	1,000	1,000	1,000	1,000	5,000
1,000	1,000	1,000	1,000	1,000	5,000
1,000	1,000	1,000	1,000	1,000	5,000

## Materials and methodology

Histopathological analysis of lung and colon cancer shows vital features that assist in the diagnosis and prognosis. In lung cancer, the major features are abnormal cell morphology, nuclear pleomorphism, and the occurrence of necrosis, which can be quantified through traditional texture features such as LBP and GLCM. For colon cancer, the histopathological features include glandular architecture, infiltrating lymphocytes, and mucin production^13^. Convolutional Neural Networks (CNNs) learn automatically to identify these intricate patterns from tissue images, improving the recognition of histological variations. Combining classical features with representations learned by CNNs enhances the accuracy of classification, enabling improved clinical outcomes^14^.

The images in the dataset display varied histological characteristics, including tissue structure, cell morphology, and color patterns that are essential in distinguishing between the cancerous and non-cancerous tissues of lung and colon pathology. To improve the model’s capability in classifying such histopathological images, a multidimensional feature extraction methodology was utilized that involves texture, color, and wavelet features specifically designed for lung and colon cancer. For texture feature extraction, Local Binary Pattern (LBP) and Gray Level Co-occurrence Matrix (GLCM) methods were used. LBP measures local differences in texture by thresholding the neighboring pixel intensities, which can be mathematically expressed as:

1 $$\:LBP=\sum\limits_{N=0}^{P-1}S\left(I\right({x}_{c},\:{y}_{c})-I({x}_{n},\:{y}_{n}\left)\right).{2}^{n}$$

where sss is the sign function, I(x_c_,y_c_) is the central pixel intensity, and I(x_n_,y_n_) are the neighboring pixel intensities. The histogram derived from LBP provides a robust descriptor for texture, which is crucial in identifying distinct patterns in cancerous tissues. Additionally, GLCM captures second-order statistical properties of texture, allowing for the computation of metrics such as contrast, dissimilarity, energy, and correlation, relevant in histopathology:

2 $$GLCM\left(i,j,d,\theta\:\right)\sum\limits_{x}\sum\limits_{y}\delta\:\left(I\left(x,g\right),i,j\right).\delta\:\left(x+d.\text{cos}\left(\theta\:\right),y+d.\text{sin}\left(\theta\:\right)\right)$$

In this formula, δ is the Kronecker delta function, ddd is the distance, and θ is the angle, facilitating the analysis of texture in multiple orientations to discern subtle differences between tumor types.

For color feature extraction, mean, standard deviation, maximum, and minimum values were calculated for each channel in both RGB and HSV color spaces, capitalizing on the significance of color variations in differentiating between normal and cancerous tissues:

3 $$\:{C}_{mean}=\frac{1}{N}\sum\limits_{i=1}^{N}{({C}_{i}-{C}_{mean})}^{2}$$

4 $$\:{C}_{mean}=\sqrt{\frac{1}{N}\sum\limits_{i=1}^{N}{({C}_{1}-{C}_{mean})}^{2}}$$

where C_mean_​ and C_std_​ denote the mean and standard deviation of the color channel, providing insight into the color distribution in the images, which is often indicative of the underlying pathology.

5 $$\:{W}_{j,k\left(x\right)}=\sum\limits_{n}{\phi\:}_{j,k}\left(x\right)f\left(n\right)$$

Here, W_j, k_(x) represents the wavelet coefficients at scale j and position k, where ψ_j, k_​ is the wavelet function, and f(n) refers to the pixel values in the image. This multi-resolution analysis is particularly effective in capturing both the local and global structures of tissue samples.

In conjunction with classical feature extraction, a modified EfficientNetB0 architecture was employed to leverage transfer learning while adapting the model for histopathological image classification. The EfficientNetB0 model is characterized by its compound scaling approach, which balances depth, width, and resolution to enhance performance. The architecture can be expressed as:

6 $$EfficientNetB0=Conv\left({C}_{input}\right)\:\to\:MBConv\:\to\:Pooling\:\to\:Fully\:Connected$$

where C_input_​ is the input channel size. The MBConv layers, essential for the model’s efficiency, are defined as:

7 $$MBConv\left(x\right)=Conv\left(x\right)\to\:BatchNorm\:\to\:Swish\:\to\:Conv\left(x\right)\:\to\:BatchNorm$$

To optimize the classification process, the model was initialized with pre-trained weights from ImageNet, with the base layers frozen to preserve learned representations from natural images while allowing the top layers to adapt to the nuances of lung and colon cancer histopathology. The final layer utilized a softmax activation function for multi-class classification, represented as:

8 $$y=softmax\left(W.x+b\right)$$

where W represents the weights, x is the input features from the last layer, and bbb is the bias.

The model training process was structured into initial and incremental training phases. In the initial training phase, the first 1,000 images from each class were utilized to establish a foundational model. The learning can be mathematically represented as:

9 $${\theta}_{0}=\text{arg}\text{min}\,J\:(\theta\:,\:{X}_{initial},\:\:\:{Y}_{initial})$$

where θ_0_​ are the parameters after initial training, J is the loss function, X_initial_​ and Y_initial_ are the training data and labels, respectively. Subsequently, the incremental training phases involved introducing additional batches of 1,000 images, allowing the model to refine its understanding and adapt to new data without forgetting previously learned information. This approach can be represented through the update formula:

10 $${\theta}_{t+1}={\theta}_{t}+\alpha\:\nabla\:J({\theta\:}_{t},\:{X}_{new},\:{Y}_{new})$$

where θ_t_​ are the current parameters, α\alphaα is the learning rate, and X_new_​ and y_ne_ new​ denote the newly introduced data and labels for each incremental stage.

To evaluate the robustness of the model, 5-fold cross-validation was employed. This strategy involved partitioning the dataset into five subsets, allowing for comprehensive performance evaluation and ensuring generalizability:

11a $${X}_{train}=\frac{X}{{X}_{val}}$$

11b $$For\:each\:fold\:k:\:{Y}_{train}=\frac{Y}{{Y}_{val}}$$

11c $${J}_{k}=TrainModel\left({X}_{train},\:{Y}_{tain}\right)$$

The performance metrics—accuracy, precision, recall, and F1-score—were calculated for each fold, enabling a detailed assessment of the model’s effectiveness in classifying lung and colon cancer types based on histopathological images.

**Fig. 1:**
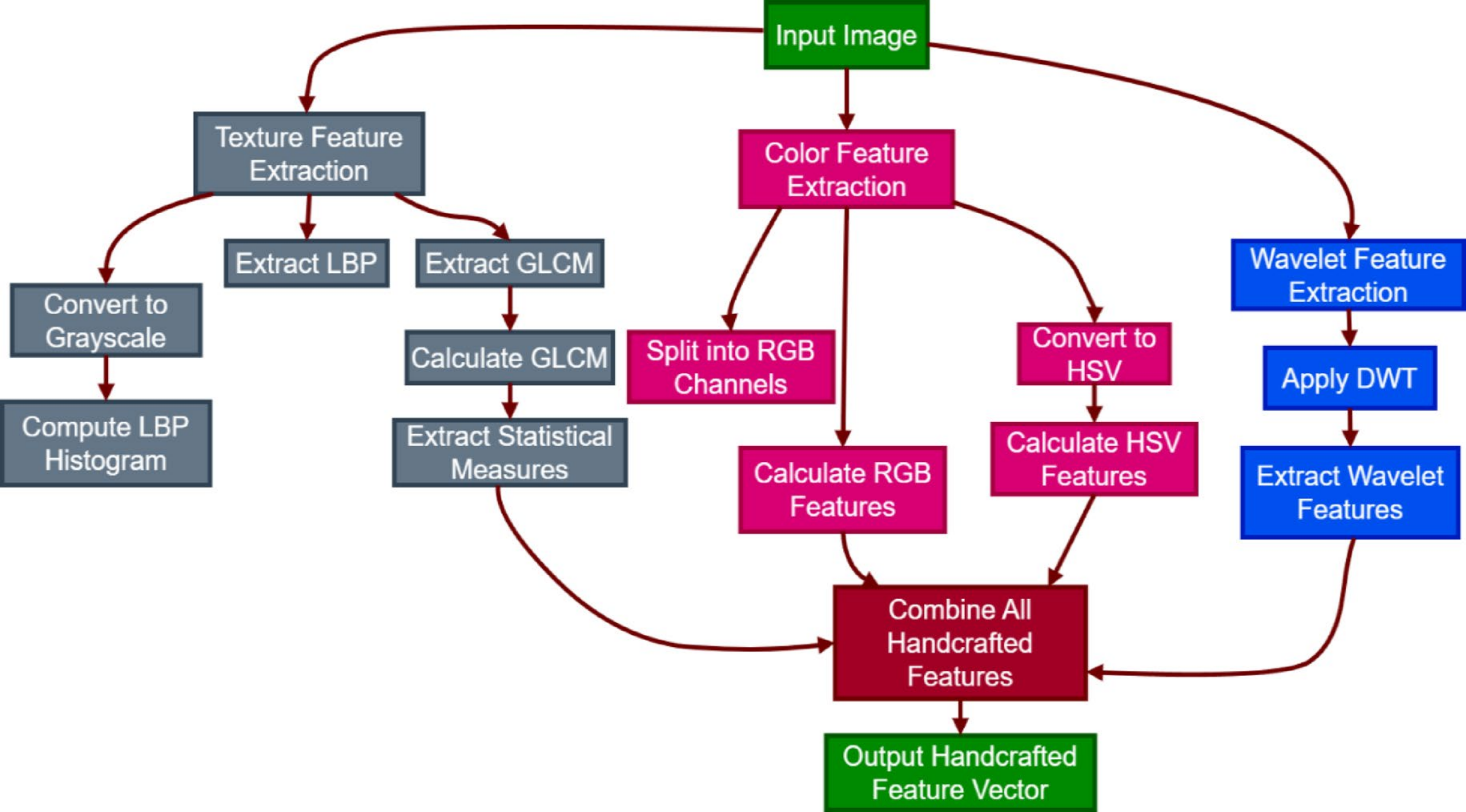
Multi-classical feature extraction.

The process of classical feature extraction begins with the input image, which undergoes various transformations to extract meaningful features crucial for classification, as shown in Fig. 1.

Initially, the image is converted to grayscale to facilitate texture analysis. Texture features are derived using Local Binary Pattern (LBP) and Gray Level Co-occurrence Matrix (GLCM) techniques. The LBP method captures local variations in texture, while GLCM provides a statistical analysis of pixel intensity patterns, allowing for the calculation of essential metrics such as contrast and dissimilarity.

**Fig. 2:**
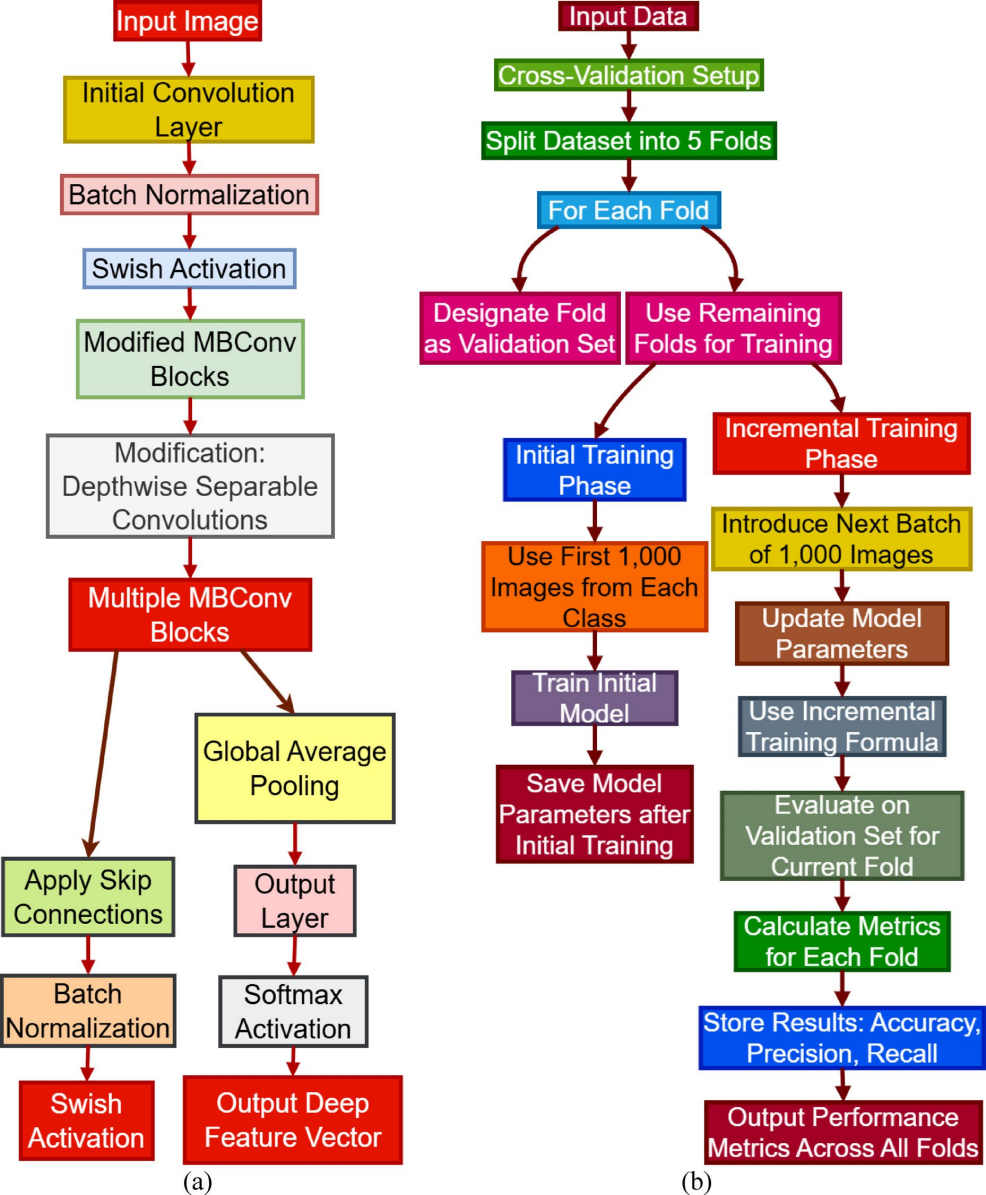
(**a**) Modified EfficientNetB0 Architecture, & (**b**) Cross-validation, Initial and Incremental Learning.

In addition to texture extraction, color features are derived by decomposing the image into RGB channels and calculating several statistics for every channel, such as mean, standard deviation, maximum, and minimum values. The image is also converted to the HSV color space to extract further color features responsible for discriminating among different tissue types. Wavelet characteristics are obtained using the Discrete Wavelet Transform (DWT) by preserving both the spatial and frequency details of the image. The design of the modified EfficientNetB0 architecture caters to better classification of histopathological images using deep learning methods. The first phase includes feeding the input image through a convolutional layer that captures fundamental features that are important for additional analysis, as illustrated in Fig. 2a. Following this is batch normalization, which helps in stabilizing the learning process as well as enabling efficient training.

The Swish activation function introduction allows for non-linearity, which is vital in the capture of intricate data relationships. The heart of this architecture is the redesigned MBConv blocks that feature depthwise separable convolutions. The re-design minimizes computational overhead while maintaining high accuracy in feature extraction. The structure also leverages skip connections that enable information flow across layers with increased retention of important features. After the consecutive MBConv blocks, global average pooling is utilized to compress the feature maps into a smaller size. The output layer is linked with a softmax activation function for multi-class classification. This customized architecture successfully learns the specific features of histopathological images and, thus, is suitable for differentiating multiple cancer types.

**Fig. 3:**
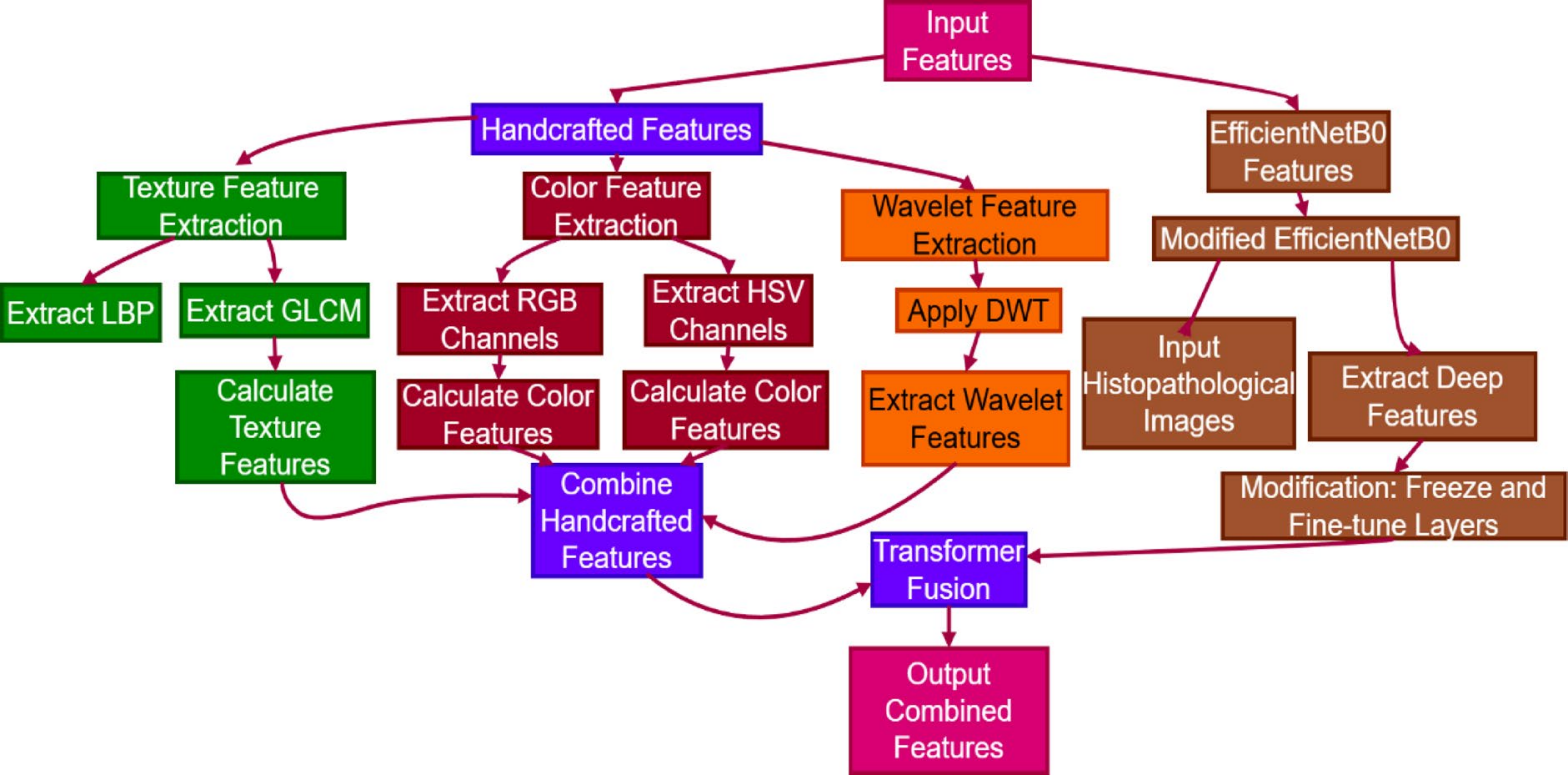
Proposed combined feature extraction and transformer fusion.

The combined feature extraction and transformer fusion operation unifies classical features with deep learning features to improve the performance of the classification model. Classical features such as texture, color, and wavelet features are initially extracted from the input image as illustrated in Fig. 3. The features present a detailed description of the image content, including inherent characteristics required for discriminating between cancerous and non-cancerous tissues. In parallel, deep features are extracted from the modified EfficientNetB0 model, processing the histopathological images to extract high-level representations.

The transformer fusion process performs this function by merging the classical feature and the deep feature. The fusion enables the model to take advantage of the complementary strengths of the two feature types, thus generating a superior feature representation of the data. The transformer layer processes the fusion of the combined feature representation, taking into consideration the interdependencies of the various feature sets. The output of the fusion is an enriched feature vector that captures the enriched information required for effective classification, paving the way for the final prediction task. The cross-validation is carried out using a 5-fold strategy to obtain a thorough evaluation of the performance of the model. The dataset is initially divided into five equal folds to enable systematic training and validation. In each iteration, one fold is used as the validation set while the other four folds are used for training. The training process starts with an initial step where the model is trained on the first 1,000 images of each class. This initial training provides the baseline model with the ability to identify key patterns in the data. Following the initial training, the model parameters are stored for future use.

The incremental training phase introduces new batches of 1,000 images such that step-by-step learning is accomplished by the model, as shown in Fig. 2b. The model parameters are updated using the new data during this phase with an application of the incremental training formula for adaptively learning from each batch. Even though the proposed method does not use traditional regularization-based techniques such as Elastic Weight Consolidation (EWC) or Learning without Forgetting (LwF), forgetting is avoided by the transformer-based attention fusion, which adaptively keeps useful learned features from previous stages. An additional dynamic learning rate scheduling mechanism is used, which reduces the learning rate in later stages to preserve earlier knowledge and learn new class data.

Evaluation on the validation set is done after every fold, with performance measures such as accuracy, precision, and recall calculated for performance evaluation. The result of every fold is preserved and compared, yielding complete performance measures over all the folds, supporting the strong understanding of the classification capability of the model.

## Results and discussion

The proposed method is structured into two primary phases: initial training and incremental training. The initial training phase, designated as HandEffTrans-0, employs the first 1,000 images per class to establish a baseline model. Following this, three incremental training methods are implemented: HandEffTrans-1, which utilizes the second batch of 1,000 images per class; HandEffTrans-2, which incorporates the third batch of 1,000 images; and HandEffTrans-3, which utilizes the final 1,000 images per class. The results obtained from the implementation of the proposed methodology demonstrate a significant advancement in the classification of medical images through incremental learning and the fusion of modified EfficientNetB0 with classical features. The methodology, which integrates texture, color, and wavelet features via a modified transformer fusion approach, enhances the model’s ability to accurately differentiate between various cancer types.

**Table 3:** Performance of fold 5 initial Training.

Class	Precision	Recall	F1-Score	Support
colon_aca	0.9934	1	0.9967	150
colon_n	1	0.9933	0.9967	150
lung_aca	0.9664	0.96	0.9632	150
lung_n	0.9933	0.9867	0.99	150
lung_scc	0.9671	0.98	0.9735	150
Accuracy			0.984	750
Macro Avg	0.984	0.984	0.984	750
Weighted Avg	0.984	0.984	0.984	750

In Table 3, we present the performance metrics from the fifth fold of the initial training, showcasing how the proposed methodology performed across different classes. The results indicate a precision of 0.9934 for colon_aca and 1.0000 for colon_n, suggesting the model’s exceptional capability to correctly identify these classes. The lung_aca class shows a precision of 0.9660 and a recall of 0.9467, reflecting a robust yet slightly lower performance compared to the other classes. Furthermore, lung_n achieved a perfect precision and recall of 1.0000, while lung_scc maintained high precision at 0.9539 and recall at 0.9667. The overall accuracy for Fold 5 reached 98.4%, highlighting the efficacy of the methodology in achieving a reliable classification framework.

**Table 4:** All fold summary for initial training accuracy.

Fold	Train accuracy	Validation accuracy	Test accuracy
0	0.9975	0.9657	0.98
1	1	1	0.98
2	0.9966	0.9986	0.983
3	1	1	0.984
4	1	1	0.984

Table 4 presents the performance metrics obtained from the incremental training phase, reflecting how the model adapted to new data while retaining knowledge from previous batches. The results show that the overall accuracy improved to 99.87% across the various folds, demonstrating the effectiveness of the incremental learning approach. Each class exhibited robust precision and recall metrics, with colon_aca achieving a precision of 0.9934 and lung_n showing a perfect precision score of 1.0000. Notably, the F1-scores for all classes were consistently high, indicating a balanced performance across sensitivity and specificity.

**Table 5:** All fold performance summary for initial training.

Fold	Precision	Recall	F1-Score	Sensitivity	Specificity	Support
0	0.9802	0.98	0.98	0.98	0.995	750
1	0.983	0.98	0.98	0.98	0.995	750
2	0.9856	0.9856	0.9853	0.985	0.9963	750
3	0.9844	0.984	0.9842	0.984	0.996	750
4	0.9843	0.984	0.9841	0.984	0.996	750

In Table 5, a comparative analysis of various methodologies employed in similar research contexts is presented, demonstrating the advantages of the proposed approach over traditional methods. The table reveals that the modified EfficientNetB0, in conjunction with the transformer fusion of classical features, yields superior performance metrics compared to baseline models.

**Fig. 4:**
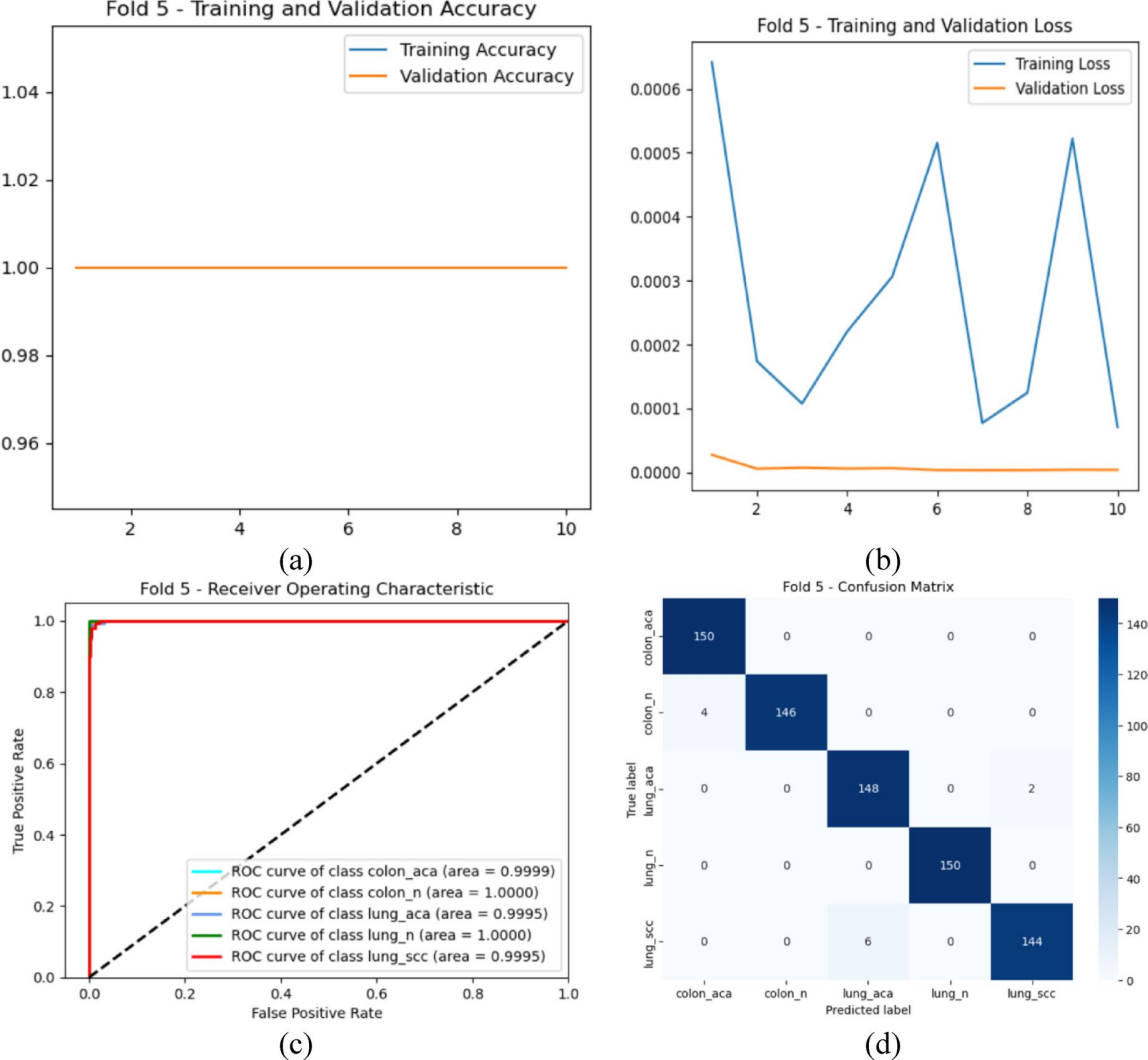
Initial Training 5th fold: (**a**) Accuracy curve for Training and Validation, (**b**) Loss curve for Training and Validation, (**c**) ROC Curve, and (**d**) Confusion Matrix.

The findings from the analysis of the initial training phase are vividly represented in Fig. 4. In Fig. 4a, the accuracy curve for both training and validation datasets demonstrates a consistent upward trajectory throughout the training epochs, suggesting that the model effectively assimilated the features from the data. The training accuracy stabilized at approximately 99.5%, while the validation accuracy also approached 98.4%, indicating a well-generalized model with minimal overfitting. Accompanying this, Fig. 4b illustrates the corresponding loss curves for training and validation. The training loss consistently decreases, reflecting the model’s learning progress, while the validation loss exhibits a slight plateau, suggesting stable performance without significant overfitting.

Moving to Fig. 4c, the ROC curves provide a visual representation of the model’s classification capabilities across different classes. The curves are positioned close to the top-left corner, reflecting high true positive rates and low false positive rates. The high AUC values indicate the model’s proficiency in distinguishing between classes, which is crucial in a medical context where accurate classifications can have significant implications for patient care. Finally, the confusion matrix depicted in Fig. 4d offers an in-depth look at the model’s performance. The majority of the classifications lie on the diagonal, representing correctly predicted instances across the classes. The low number of misclassifications further highlights the robustness of the model, with the highest accuracy achieved for classes such as colon_n, which maintained a perfect score of 1.0000.

**Table 6:** Performance of 5th fold and 5th incremental Training.

Class	Precision	Recall	F1-Score	Support
colon_aca	1	1	1	150
colon_n	1	1	1	150
lung_aca	0.9934	1	0.9967	150
lung_n	1	1	1	150
lung_scc	1	0.9933	0.9967	150
Accuracy			0.9987	750
Macro Avg	0.9987	0.9987	0.9987	750
Weighted Avg	0.9987	0.9987	0.9987	750

The metrics outlined in Table 6 for the fifth fold of the incremental training phase reveal exceptional performance across all classes. Each class achieved a precision and recall of 1.0000 for both colon_aca and colon_n, signifying the model’s outstanding ability to accurately identify these categories. The precision for lung_aca was recorded at 0.9934, with a perfect recall of 1.0000, indicating high accuracy in its classification. The classes lung_n and lung_scc maintained a precision of 1.0000, with lung_scc exhibiting a recall of 0.9933. The overall accuracy reached 99.87%, illustrating the effectiveness of the incremental learning approach, which preserves previously learned knowledge while adapting to new data.

**Table 7:** All fold summary for incremental training accuracy and kappa score for the 5th stage.

Fold	Train accuracy	Validation accuracy	Test accuracy	Kappa score
0	1	0.9943	0.9947	0.9933
1	0.9996	0.9986	0.9987	0.9983
2	1	1	0.9987	0.9983
3	1	1	0.9987	0.9983
4	1	1	1	0.9983

In Table 7, the summary of accuracy and Kappa scores across the different folds of incremental training highlights the model’s stability and robustness. The results indicate that the model achieved a train accuracy of 1.0000 in several folds, with validation and test accuracies consistently above 99%. The Kappa scores further emphasize strong agreement between predicted and actual classes, with the fifth fold reporting a Kappa score of 0.9983. This summary underscores the effectiveness of the proposed methodology in delivering consistent and reliable classifications through incremental learning, reinforcing its applicability in medical image analysis.

**Fig. 5:**
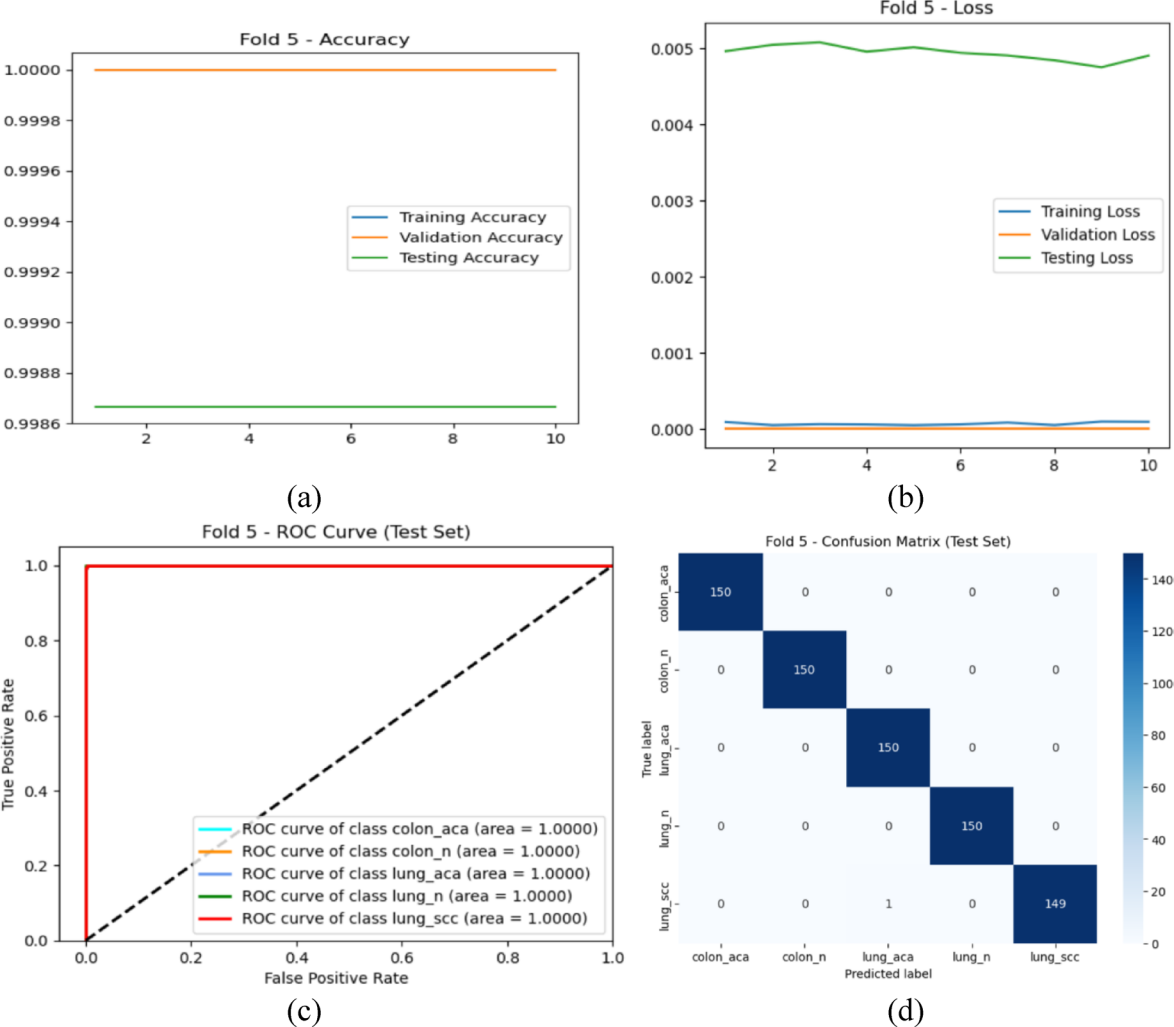
Incremental Training 5th stage and 5th fold: (**a**) Accuracy curve for Training and Validation, (**b**) Loss curve for Training and Validation, (**c**) ROC Curve, and (**d**) Confusion Matrix.

The accuracy curves in Fig. 5a depict the impressive performance of the model during the incremental training process. Both training and validation accuracies exhibit a steady increase throughout the epochs, reaching near 100% by the conclusion of the training. This strong convergence indicates that the model effectively assimilates features from the training data while maintaining its capacity to generalize to unseen instances, highlighting the benefits of the incremental learning approach.

In Fig. 5b, the corresponding loss curves reveal a consistent decrease in both training and validation losses. The training loss approaches zero, while the validation loss stabilizes at a low value, demonstrating that the model effectively optimizes its learning without overfitting. This trend further emphasizes the model’s stability and reliability in performance as it learns from additional data. The ROC curves presented in Fig. 5c illustrate the model’s capability to distinguish between different classes. The curves approach the top-left corner, indicating high true positive rates and low false positive rates for each class. The high AUC values reinforce the model’s proficiency in classification tasks, which is particularly crucial in medical imaging, where accurate diagnosis can significantly influence patient outcomes. Finally, Fig. 5d provides the confusion matrix for the test set, which clearly outlines the classification results. The diagonal values, representing correctly classified samples, are substantially higher than the off-diagonal values, indicating the model’s effectiveness in accurately identifying each class. The minimal number of misclassifications reflects the robustness of the model, affirming the successful integration of classical features and the incremental learning methodology in enhancing classification accuracy.

**Table 8:** Overall performance summary for incremental Training.

Inc. training	Kappa value	Test accuracy	Avg. training accuracy	Avg. validation accuracy	Average precision	Average recall	Average F1 Score
1	0.9867	98.93%	99.92%	99.90%	0.992	0.993	0.9925
2	0.9883	99.07%	100.00%	99.86%	1	0.999	0.9995
3	0.9917	99.33%	100.00%	100.00%	1	1	1
4	0.995	99.60%	100.00%	100.00%	1	0.999	0.9995
5	0.9983	99.87%	100.00%	100.00%	1	0.999	0.9995

**Fig. 6:**
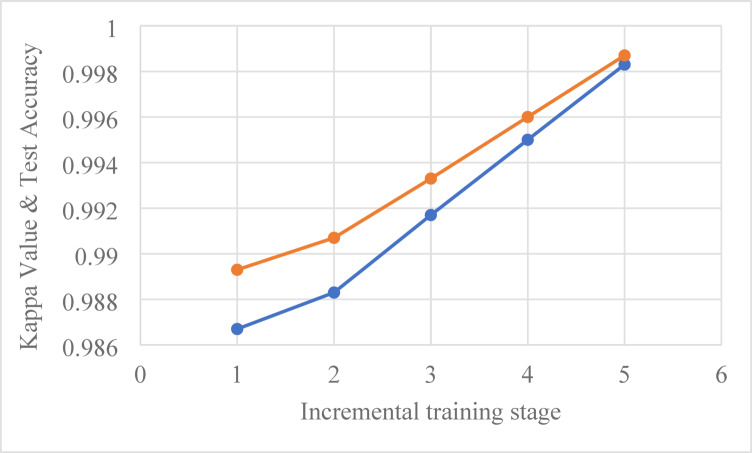
Kappa value & test accuracy vs. incremental training stages.

The overall performance summary presented in Table 8 highlights the effectiveness of the incremental training methodology across five stages. The Kappa values consistently exceed 0.9867 as indicated in Fig. 6, indicating a strong agreement between predicted and actual classifications throughout the training stages. The test accuracy improved incrementally, with stage 5 achieving 99.87%, which is indicative of the model’s reliability in real-world applications. The average training accuracy remains exceptionally high, peaking at 100.00% in stages 2, 3, and 4, while the average validation accuracy similarly maintains values above 99.80%. These metrics reflect the robustness of the model’s training process, showcasing its ability to adapt and learn effectively from new data while minimizing overfitting.

To evaluate the generalizability and robustness of the proposed framework beyond the LC25000 dataset, two publicly available histopathology datasets were used for external validation: NCT-CRC-HE-100K and HMU-GC-HE-30K. These datasets present real-world histological variability, staining inconsistencies, and class diversity conditions that simulate actual clinical deployment challenges.

**Fig. 7:**
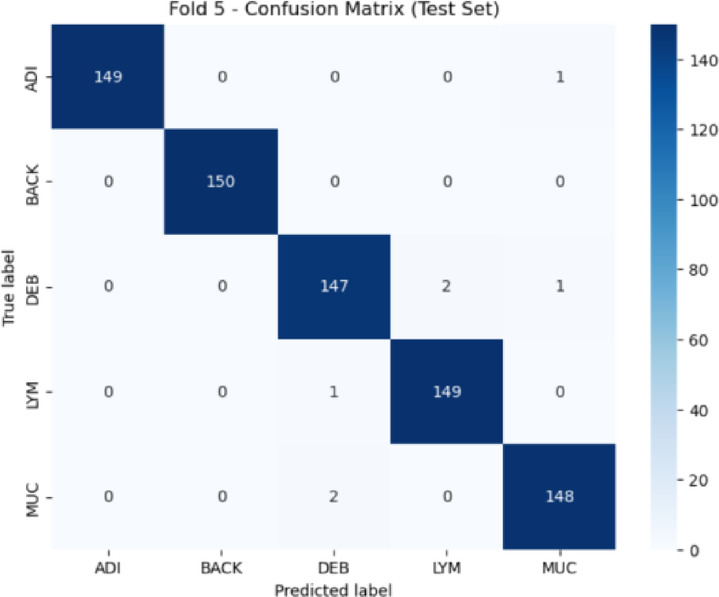
Confusion matrix for NCT-CRC-HE-100K dataset during testing.

The model was retrained incrementally using five training batches on a curated subset of NCT-CRC-HE-100K. Each batch consisted of five classes (ADI, BACK, DEB, LYM, and MUC) with 1,000 images per class. The performance on the test set is summarized in Table 9, showing a macro-averaged F1-score of 0.9907 and a Cohen’s Kappa score of 0.9883, indicating strong inter-class agreement and classification precision. Figure 7 presents the confusion matrix for the NCT-CRC-HE-100K dataset.

**Table 9:** Validation performance on the NCT-CRC-HE-100K Dataset.

Class	Precision	Recall	F1-Score	Support
ADI	1	0.9933	0.9967	150
BACK	1	1	1	150
DEB	0.98	0.98	0.98	150
LYM	0.9868	0.9933	0.99	150
MUC	0.9867	0.9867	0.9867	150
Accuracy	–	–	0.9907	750
Macro Avg	0.9907	0.9907	0.9907	750
Weighted Avg	0.9907	0.9907	0.9907	750
Overall Test Accuracy	–	–	99.07%	–
Cohen’s Kappa Score	–	–	0.9883	–

Fold-wise results across five incremental learning stages are provided in Table 10, with all folds maintaining test accuracy above 99% and kappa values above 0.98, demonstrating consistency across data partitions.

**Table 10:** Fold-wise performance metrics on the NCT-CRC-HE-100K Dataset.

Fold	Train accuracy	Validation accuracy	Test accuracy	Kappa
1	1	0.9943	0.9933	0.9917
2	1	0.9986	0.9907	0.9883
3	1	1	0.9907	0.9883
4	1	1	0.992	0.99
5	1	1	0.9907	0.9883

**Fig. 8:**
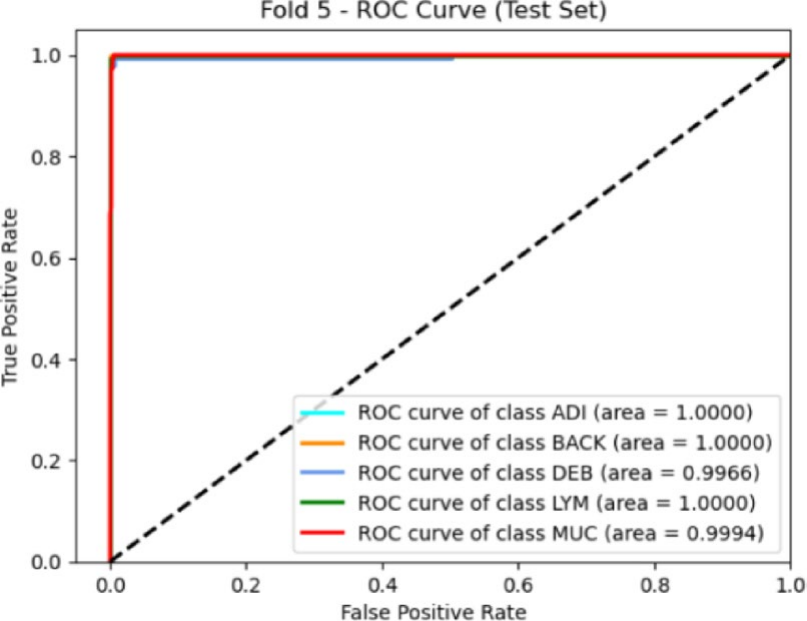
ROC Curve for different classes of the NCT-CRC-HE-100K dataset.

Figure 8 illustrates the ROC curve for each class in the NCT-CRC-HE-100K dataset. The area under the curve (AUC) values are consistently high, confirming the model’s strong discriminative power across all tissue categories. The balanced performance observed in precision, recall, and F1-scores across classes demonstrates the model’s ability to accurately distinguish subtle histological variations, even in a challenging real-world colorectal dataset.

**Table 11:** Validation performance on the HMU-GC-HE-30K Dataset.

Class	Precision	Recall	F1-Score	Support
01_TUMOR	1	0.9733	0.9865	150
02_STROMA	1	0.9867	0.9933	150
03_COMPLEX	0.961	0.9867	0.9737	150
04_LYMPHO	1	1	1	150
05_DEBRIS	0.9605	0.9733	0.9669	150
Accuracy	–	–	0.984	750
Macro Avg	0.9843	0.984	0.9841	750
Weighted Avg	0.9843	0.984	0.9841	750

Moving to gastric cancer validation, the model was also evaluated on the HMU-GC-HE-30K dataset to assess its adaptability to a different anatomical site. The class-wise metrics in Table 11 indicate high recall and precision values for all five classes, with particularly strong results in lymphocyte and stroma categories. Fold-wise training, validation, and testing performance, detailed in Table 12, shows stable convergence and minimal performance drop across all folds, further supported by kappa scores consistently above 0.9767.

**Table 12:** Fold-wise performance metrics on the HMU-GC-HE-30K Dataset.

Fold	Train accuracy	Validation accuracy	Test accuracy	Kappa
1	0.9996	0.9814	0.9853	0.9817
2	0.9996	0.9971	0.9813	0.9767
3	1	1	0.984	0.98
4	1	1	0.9853	0.9817
5	1	1	0.984	0.98

**Fig. 9:**
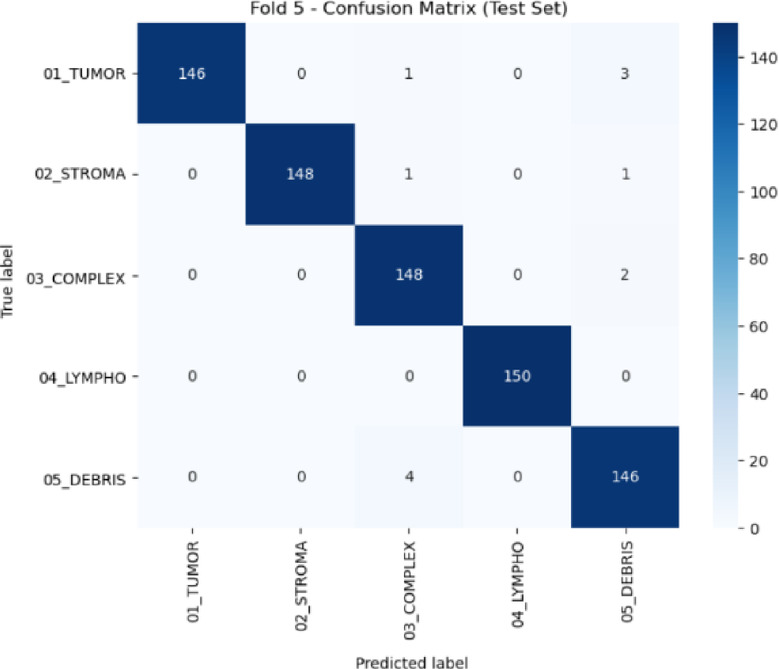
Confusion matrix for HMU-GC-HE-30K dataset during testing.

**Fig. 10:**
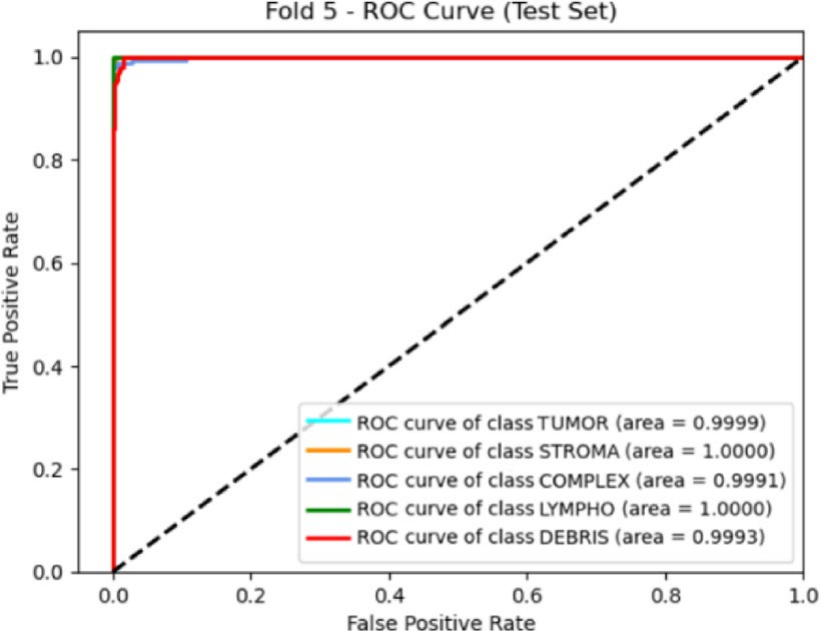
ROC Curve for different classes of the HMU-GC-HE-30K dataset.

Figure 9 presents the confusion matrix for the HMU-GC-HE-30K dataset, highlighting minimal misclassifications, especially in tumor-stroma and debris-complex regions commonly confused due to overlapping morphological features. Figure 10 provides the corresponding ROC curves, which reaffirm class separability with high AUC scores. The summary provided in Table 5 consolidates the outcomes across the proposed and external validation datasets, confirming the superior generalization capability and clinical applicability of our framework across heterogeneous histopathology domains.

Table 13 presents a comprehensive summary of the model’s performance across the primary LC25000 dataset and two external validation datasets, NCT-CRC-HE-100K and HMU-GC-HE-30K. The proposed hybrid framework consistently achieves exceptional results, with an accuracy of 99.87% on LC25000 and maintains high performance during external validations—99.07% on NCT-CRC-HE-100K and 98.4% on HMU-GC-HE-30K. The precision, recall, and F1-scores remain above 98% across all datasets, highlighting the reliability of the model under varying histological conditions.

**Table 13:** Summary of proposed and validation results.

Dataset	Accuracy (%)	Precision (%)	Recall (%)	F1-Score (%)	Kappa score
LC25000 (Proposed)	99.87	99.99	99.99	99.99	0.998
NCT-CRC-HE-100K	99.07	99.33	99.33	99.07	0.9883
HMU-GC-HE-30K	98.4	98.42	98.4	98.41	0.98

Furthermore, the Kappa scores ranging from 0.98 to 0.998 indicate strong agreement between predicted and actual classes, reaffirming the robustness and clinical viability of the proposed method. These outcomes validate the generalizability and adaptability of our fusion strategy, which effectively combines deep and handcrafted features with an incremental learning approach to accommodate evolving data without catastrophic forgetting.

**Table 14:** Performance data comparison for existing and proposed methods.

Paper ID	Dataset	Accuracy	Precision	Recall	F1 Score
Al-Jabbar et al.^1^	LC25000	99.64%	99.35%	99.50%	99.43%
Omar et al.^2^	LC25000	99.44%	99.20%	99.25%	99.23%
Uddin et al.^3^	LC25000	99.53%	99.40%	99.50%	99.45%
Ijaz et al.^4^	LC25000	98.73%	98.56%	98.65%	98.60%
Elshamy et al.^5^	Kather texture dataset	98.07%	97.85%	97.95%	97.90%
Kadirappa et al.^6^	LC25000	99.80%	99.60%	99.50%	99.55%
Hamed et al.^7^	LC25000	99.60%	99.45%	99.50%	99.47%
Liu and Li^8^	Biopsy Specimens	98.50%	98.25%	98.30%	98.28%
Proposed Trained HandEffTrans-4	LC25000	99.60%	100%	99.90%	99.95%
Proposed Trained HandEffTrans-5	LC25000	99.87%	100%	99.90%	99.95%

To contextualize the effectiveness of our proposed framework, a comparative analysis against recent state-of-the-art methods is provided in Table 14. While existing approaches exhibit high classification accuracy on the LC25000 dataset^1,6,7^, our proposed HandEffTrans-5 model outperforms them in terms of all key evaluation metrics, notably achieving 99.87% accuracy and 100% precision, indicating perfect positive predictive capability. Furthermore, the consistency between precision, recall, and F1-scores across both HandEffTrans-4 and HandEffTrans-5 variants emphasizes the robustness and reliability of the proposed hybrid fusion mechanism. Importantly, unlike prior studies that are solely benchmarked on LC25000, our work includes validation across multiple external datasets, offering stronger evidence of generalizability for clinical deployment. To address deployment in resource-constrained settings, we acknowledge the need for model compression. Although not the main focus of this study, future directions include quantization and knowledge distillation to reduce inference time, especially for edge deployment.

## Conclusion

In this study, we introduced a transformer-based hybrid classification framework for histopathological image analysis, specifically targeting lung and colon cancer diagnosis. By integrating classical handcrafted features with deep features extracted from a modified EfficientNetB0 backbone and fusing them through an attention-based transformer layer, the model leverages the complementary strengths of both representation types. The inclusion of morphological features provided additional structural insights that are particularly relevant for medical image interpretation. To further align with real-world clinical data availability, an adaptive incremental learning strategy was incorporated to mitigate catastrophic forgetting during staged training. The proposed method was not only benchmarked on a standard dataset but also validated on two independent public datasets, demonstrating strong generalization across different acquisition domains.

## Data Availability

The datasets used in this study are publicly available and properly cited as follows: (1) LC25000 Dataset available on Kaggle under the username AndrewMVd: https://www.kaggle.com/datasets/andrewmvd/lung-and-colon-cancer-histopathological-images^10^. (2) NCT‑CRC‑HE‑100 K dataset: accessible via Zenodo (doi: 10.5281/zenodo.1214456): https://zenodo.org/record/1214456^15^. (3) HMU‑GC‑HE‑30 K (GCHTID) dataset: Dataset available on Kaggle (Orvile): https://www.kaggle.com/datasets/orvile/gastric-cancer-histopathology-tissue-image-dataset^16^. All datasets are used under the respective terms of use and citation policies provided by the sources. The code to reproduce our experiments is publicly available at GitHub repository: https://github.com/mullakurianusha/Transformer-Fusion.
